# Cooperation between liver-specific mutations of *pten* and *tp53* genetically induces hepatocarcinogenesis in zebrafish

**DOI:** 10.1186/s13046-021-02061-y

**Published:** 2021-08-20

**Authors:** Juanjuan Luo, Chunjiao Lu, Meilan Feng, Lu Dai, Maya Wang, Yang Qiu, Huilu Zheng, Yao Liu, Li Li, Bo Tang, Chuan Xu, Yajun Wang, Xiaojun Yang

**Affiliations:** 1 Key laboratory of Bio-resources and Eco-environment of Ministry of Education, College of Life Science, Sichuan University, Chengdu, China; 2 Shantou University Medical College, Shantou, China; 3 Key Laboratory of Freshwater Fish Reproduction and Development, Ministry of Education, Key Laboratory of Aquatic Science of Chongqing, Laboratory of Molecular Developmental Biology, School of Life Sciences, Southwest University, Chongqing, China; 4 Department of Hepatobiliary Surgery, The first Affiliated Hospital of Guangxi Medical University, Nanning, China; 5 Integrative Cancer Center & Cancer Clinical Research Center, Cancer Center, Sichuan Cancer Hospital & Institute Sichuan, School of Medicine University of Electronic Science and Technology of China, Chengdu, 610041 China

**Keywords:** Pten, Tp53, Akt, Hepatocellular carcinoma, Zebrafish

## Abstract

**Background:**

Liver cancer, mainly hepatocellular carcinoma, is one of the deadliest cancers worldwide and has a poor prognosis due to insufficient understanding of hepatocarcinogenesis. Previous studies have revealed that the mutations in *PTEN* and *TP53* are the two most common genetic events in hepatocarcinogenesis. Here, we illustrated the crosstalk between aberrant Pten and Tp53 pathways during hepatocarcinogenesis in zebrafish.

**Methods:**

We used the CRISPR/Cas9 system to establish several transgenic zebrafish lines with single or double tissue-specific mutations of *pten* and *tp53* to genetically induce liver tumorigenesis. Next, the morphological and histological determination were performed to investigate the roles of Pten and Tp53 signalling pathways in hepatocarcinogenesis in zebrafish.

**Results:**

We demonstrated that Pten loss alone induces hepatocarcinogenesis with only low efficiency, whereas single mutation of *tp53* failed to induce tumour formation in liver tissue in zebrafish. Moreover, zebrafish with double mutations of *pten* and *tp53* exhibits a much higher tumour incidence, higher-grade histology, and a shorter survival time than single-mutant zebrafish, indicating that these two signalling pathways play important roles in dynamic biological events critical for the initiation and progression of hepatocarcinogenesis in zebrafish. Further histological and pathological analyses showed significant similarity between the tumours generated from liver tissues of zebrafish and humans. Furthermore, the treatment with MK-2206, a specific Akt inhibitor, effectively suppressed hepatocarcinogenesis in zebrafish.

**Conclusion:**

Our findings will offer a preclinical animal model for genetically investigating hepatocarcinogenesis and provide a useful platform for high-throughput anticancer drug screening.

**Supplementary Information:**

The online version contains supplementary material available at 10.1186/s13046-021-02061-y.

## Background

Liver cancer is the sixth most common cancer and the third leading cause of cancer-related mortality worldwide [1]. Hepatocellular carcinoma (HCC) accounts for over 90% of primary liver cancers [2]. It generally has a poor prognosis, as it is often diagnosed at a late stage; thus, even with treatment, HCC has a high recurrence rate after resection, and a lack of curative therapies for advanced-stage disease [3]. In addition, HCC is characterized by its heterogeneity and multistage process of tumour development [4], leading to the low effectiveness of systemic chemotherapy [5]. Therefore, it is important to clarify the fundamental processes underlying HCC progression and to identify the differences among HCC subgroups, which might facilitate the appropriate treatment of patients with HCC.

Previous reports indicated that multiple risk factors, including infection with hepatitis B virus (HBV) or hepatitis C virus (HCV), chronic alcohol consumption, aflatoxin contamination, and metabolic disease, are highly correlative with HCC tumorigenesis [6–8]. These factors ultimately play critical roles in regulating multiple oncogenes or tumour suppressor genes to activate tumorigenesis-related signalling pathways [9, 10]. In humans, epigenetic alterations or gene mutations in two key signalling pathways, Akt/PTEN and TP53 pathways, have been determined to be critical in most HCC patients [11, 12]. In over 40% of diagnosed patients, primary HCC results from activation of Akt signalling or impaired expression of PTEN [10, 11, 13, 14], indicating that Akt/PTEN pathway plays a major role in hepatocarcinogenesis. Previous reports also showed that liver-specific mutation of *Pten* in mice results in fatty liver disease and late-onset liver cancer [15, 16]. In addition, Previous studies indicated that most gene mutations in HCC were associated principally with inactivation of TP53 and β-catenin signalling cascades [17]. HBV-related liver tumours have a higher rate (32–47%) of TP53-inactivating mutations than non-HBV-related liver tumours [18, 19]. It is known that TP53 acts as a tumour suppressor and is associated with tumorigenesis in most malignancies [20]. *TP53* mutation in HCC patients from Western countries are also linked with worse clinical stage and prognosis [18]. Although several previous studies investigated the potential roles of PTEN and TP53 in HCC [21, 22], the crosstalk between PTEN and TP53 signalling pathways and the dynamic histological features involved in the initiation and development of HCC require further exploration.

In the past decade, the zebrafish has been increasingly recognized as an alternative vertebrate model for studying cancer susceptibility and carcinogenesis because of the economy of zebrafish husbandry, the potential of this model for high-throughput drug screening [23], and the high similarity between the histology and gene expression profiles of zebrafish tissue and the tissues of mice and humans [24, 25]. Models of several cancers, including leukaemia, melanoma, and glioma, have been established in zebrafish [26–30]. Moreover, previous reports have indicated that several liver cancer transgenic zebrafish models have been generated. Zhen et al. showed that single transgenic expression of the fish oncogene *xmrk* or *myc* can independently induce liver cancer in zebrafish [31, 32]. In addition, liver-specific expression of oncogenic *kras*^*V12*^ can drive robust liver tumorigenesis in transgenic zebrafish [33]. A recent study also indicated that heterogeneous β-catenin activation is sufficient to cause HCC in zebrafish [34]. Another study reported that gankyrin transgenic zebrafish spontaneously developed persistent hepatocyte damage, steatosis, cholestasis, cholangitis, fibrosis, and hepatic tumours at 7–12 months of age [35]. In addition, the molecular signatures of HCC in *Xmrk*, *Kras* and *Myc* transgenic zebrafish showed high similarity with those in humans [36], suggesting that these transgenic zebrafish models might be helpful for understanding the initiation and progression of HCC. However, most previous studies mainly investigated a single oncogenic factor in hepatocarcinogenesis. Tumorigenesis and tumour progression are highly variable, and understanding the crosstalk among the multiple altered pathways, which specifically reflect features more similar to those of human disease, might be more valuable than considering each pathway separately for developing precision medicine for individual HCC patients.

As PTEN and TP53 pathways are the core signalling pathways in hepatocarcinogenesis, and although mouse models of *Tp53* and *Pten* mutation/inactivation showed that *Tp53* and *Pten* mutations can induce hepatocarcinogenesis in adult HBV transgenic mice [37], the potential roles of these signalling pathways during different stages of hepatocarcinogenesis remain poorly understood. In the present study, we developed several transgenic zebrafish lines with conditional deletion of *pten* or *tp53* gene under the control of the liver-specific *fabp10* promoter [31]. In addition, the *Cre-loxp* system, which has greatly expanded the ability to precisely investigate gene function and allows both spatial and temporal control of gene expression, was used in these transgenic fish lines. Single or double mutations induced grade I to III HCC in mature liver tissues of zebrafish with different typical phenotypes. Further investigation demonstrated that the HCC tumours derived from our zebrafish models and HCC tumours from humans exhibit molecular conservation at the morphological and histological levels. Furthermore, pharmacological blockade of Akt signalling attenuated the progression of hepatocarcinogenesis in MK-2206-treated fish, suggesting that the use of these transgenic fish models for drug screening could benefit the treatment of HCC patients.

## Materials and methods

### Fish lines, husbandry, and treatments

The wild-type (WT) zebrafish line (AB strain) was obtained from the China Zebrafish Resource Center (Wuhan, China) and raised according to the guidelines for zebrafish care [38]. Fish were maintained in aquaria with a water cycling system under a 14-h light/10-h dark cycle at 28 °C. For drug treatment, 14 days post fertilization (dpf) larvae were treated with 5 μM MK-2206 (MedChemExpress, Monmouth Junction, NJ) for 3 months. During the treatment, the tank water containing dissolved drug was refreshed daily. The animal experiments were performed according to the protocols approved by Shantou University Medical College.

### Synthesis of mRNA and gRNAs, DNA construction and microinjection

*Cas9* mRNA was generated from a pCS2 Cas9 vector by in vitro transcription using a mMESSAGE mMACHINE T7 kit (Thermo Fisher Scientific, Waltham, MA). gRNAs were designed using the CHOPCHOP website (http://chopchop.cbu.uib.no) [39]. The gRNA transcription templates were produced using T7-targetsite-F primers and the universal reverse primer gRNA-R (Supplemental Table 1), and gRNAs were transcribed using a MAXIscript T7 Kit (Thermo Fisher Scientific) and purified using a MicroElute RNA Clean-Up Kit (Omega Bio-Tek, Norcross, GA). The in vitro efficiencies of the gRNAs were determined by using a gRNA Activity Detection Kit (ViewSolid Biotech, Beijing, China).

We cloned the zebrafish *fabp10* promoter into the driver cloning vector *pTol2-CreLite* (#131783; Addgene, Watertown, MA) using the *XbaI* and *EcoRI* endonucleases (New England Biolabs, Ipswich, MA). The different gRNA sequences were inserted in the response cloning vector *Cas9-P2A-mCherry,U6:gRNA* and were then subcloned into *pTol2-Ubb-loxp-stop-loxp* with the *XhoI* and *AatII* endonucleases to construct *Ubb:Cas9-P2A-mCherry,U6:gRNA(ptena)*, *Ubb:Cas9-P2A-mCherry,U6:gRNA(ptenb), Ubb:Cas9-P2A-mCherry,U6:gRNA(tp53)*, and *Ubb:Cas9-P2A-mCherry,U6:gRNA(null)* as the control vector.

For microinjection, a mixture of the DNA construct (20 pg) and *Tol2* mRNA (20 pg) was injected into the embryos of the AB strain at the one-cell stage. For typical CRISPR experiments, a mixture of *Cas9* mRNA (600 pg) and the gRNA (25 pg) was injected into each embryo. After microinjection, the embryos were raised in E3 medium at 28 °C.

### Analysis of gRNA efficiency, T7 endonuclease I (T7E1) mutagenesis assay and the generation of tissue-specific homozygous strains

The gRNA target sites and the primer sequences used for the T7E1 assay were shown in Supplemental Table 1. To determine each specific gRNA efficiency, the total genomic DNA from 15 injected 24 h post fertilization (hpf) embryos was extracted to perform PCR amplification and T7E1 assay. PCR mixtures were produced using TransFast Taq DNA Polymerase (TransGen Biotech, Beijing, China). Thereafter, 8.5 μl of PCR product was annealed for heteroduplex formation, 1 μl of T7E1 and 0.5 ml of T7E1 buffer (ViewSolid Biotech) were then added, and the mixture was incubated at 37 °C for 25 min. The samples were further analysed by agarose gel electrophoresis. The amplified DNA from the positive mutants was inserted in the pMD19-T vector (TaKaRa Bio Inc., Japan), and 20 colonies were selected for further DNA sequencing. The gRNA efficiency was calculated as the number of mutated alleles divided by the total number of sequenced alleles.

To generation the stable F_2_ tissue-specific homozygous strains, the founder fish, which randomly integrated the specific effective gRNA into a subset of embryonic cells, outcrossed with WT fish. The mutated alleles of their F_1_ offspring were identified by using cutting-tail method, T7E1 assay and DNA sequencing at 30 dpf. The stable F_2_ tissue-specific homozygous strains, were obtained from the in-cross of F_1_ generation knockout (KO) fish lines with the same mutated alleles to ensure all hepatocytes will have identical mutation.

### Whole-mount in situ hybridization

Whole-mount in situ hybridization assays of different mutants and controls were performed as previously described [40]. The sense and anti-sense RNA probes were synthesized by in vitro transcription with a mMESSAGE mMACHINE T7 Kit (Invitrogen, Thermo Fisher Scientific, Waltham, MA). In situ hybridization was performed following our previous protocol [41].

### qPCR and immunoblotting

The dissected liver tissues from different mutant or control zebrafish were collected for total RNA extraction with TRIzol™ reagent (Invitrogen). The extracted total RNA was reverse transcribed by qRT-PCR using a QuantiTect™ SYBR Green PCR Kit (Qiagen, Valencia, CA). Relative quantification was determined by the 2^−ΔCt^ method, where Ct is the difference between the mean Ct value of the triplicates of a sample and the Ct value of the endogenous control mRNA. The sequences of the primers used in the RT-PCR assay are shown in Supplemental Table 2.

The dissected harvested liver tissues from different mutant or control zebrafish were lysed in lysis buffer (Thermo Fisher Scientific). The samples were separated by SDS-PAGE, transferred to nitrocellulose membranes (Millipore, Sigma, Burlington, MA), and immunoblotted with specific primary antibodies followed by HRP-conjugated secondary antibodies. Protein band densities were determined with a SuperSignal West Pico kit (Thermo Fisher Scientific). All information on the antibodies and the utilized concentrations is provided in Supplemental Table 3.

### Immunofluorescence, immunohistochemistry and histological examination

For immunofluorescence staining, the 25-mm cryosections of 4% paraformaldehyde-fixed sections were incubated with the primary antibodies overnight at 4 °C, and subsequently with the secondary antibodies at room temperature for 1 h. The immunostained sections were determined and photographed under a confocal microscope (FV1000; Olympus, Tokyo, Japan). In addition, the whole-embryonic fluorescent determination was performed by using an Olympus MVX10 fluorescence microscope. All information on the antibodies and the utilized concentrations is provided in Supplemental Table 3.

The randomly selected liver tissues specimens from the euthanized mutants and control fish were dissected, paraformaldehyde-fixed and paraffin-embedded for haematoxylin & eosin (H&E) staining and immunohistochemistry. After antigen retrieval and blocking, the samples were immunostained using the primary antibodies and subsequently with the appropriate secondary antibodies (Supplemental Table 3). Thereafter, the samples were then detected using avidin-bioten complex method (Dako), visualized with DAB, and evaluated with Image-Pro Plus software (Media Cybernetics Inc., Rockville, MD). The paraffin-embedded tissue microarray of human HCC specimens with the clinicopathological information (Supplemental Table 4) was obtained from Alena Biotech Ltd. (DC-Liv01020; Xi’an, China), and further confirmed by the pathologist according to the classification of Edmondson-Steiner grade and the previous described [42–45]. In addition, the histological grades of tumours generated from the liver tissues of the transgenic fish were diagnosed by the pathologist according to the previous reports [31, 33, 35, 46]. Specifically, the high-grade HCC is usually accompanied by liver hyperplasia, abnormal lipid accumulation, vascular disorders, necrosis, haemorrhage and swollen bodies, and invasion of tumour cells into blood vessels, as well as robust expression of hepatocarcinogenesis-related factors, such as phh3, Pcna and pAkt. The ethics approval was approved by the Medical Ethics Committee of Shantou University Medical College.

### Quantification and statistical analysis

All experiments were performed with the arithmetic means and standard error (SE). Student’s *t*-test for pairwise comparison or ANOVA for multivariate analysis. *P* < 0.05 was regarded as indicating statistical significance. The data were processed using SPSS statistical software version 10.0 (SPSS software Inc., Chicago, IL). All experiments were repeated at least three times with similar results.

## Results

### CRISPR/Cas9 gene editing with a guide RNA (gRNA) expression cassette induces tissue-specific *tp53* or *pten* mutation in zebrafish

**Fig. 1:**
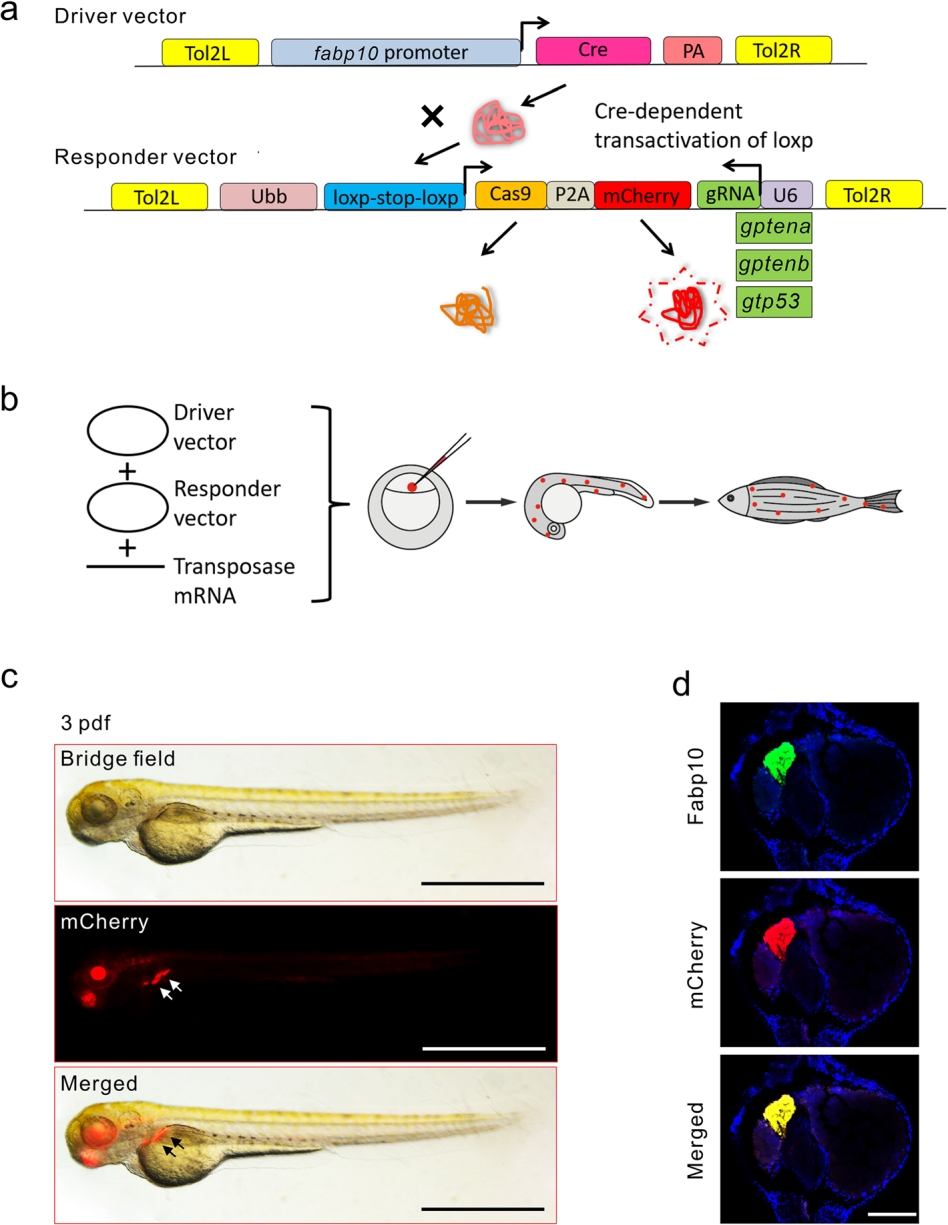
Generation of zebrafish with tissue-specific *pten* or *tp53* deficiency. **a** The driver construct provides zebrafish *fabp10* promoter-driven expression of Cre, which can bind to the *loxp-stop-loxp* cassette in the responder construct and activate the expression of *Cas9-P2A-mCherry* and the gRNA via the *fabp10* and *U6* promoters, respectively. The expression of *Cas9* can be determined by mCherry fluorescence in zebrafish larvae. The driver and responder constructs are flanked by *Tol2* transposon sites. **b** Strategy for the generation of transgenic zebrafish lines. The driver and responder constructs were mixed and injected with *Tol2* mRNA to generate mosaic founder transgenic fish lines. **c** The phenotype of the F_2_ transgenic *fabp10*^WT^ larvae at 3 dpf. Scale bars, 1 mm. **d** Colocalization of mCherry (red) and endogenous Fabp10 (green, FITC-labelled) expression in liver tissues of *fabp10*^WT^ larvae at 3 dpf. Scale bars, 400 μm

To establish *tp53* or *pten* mutation in zebrafish, CRISPR/Cas9 gene editing was performed to generate zebrafish lines with complete gene knockout [47]. We designed sequences targeting *ptena* and *ptenb*, the two homologous genes of *pten* in zebrafish, and *tp53* with CHOPCHOP (http://chopchop.cbu.uib.no/). The effective *ptena, ptenb*, and *tp53* gRNAs were identified by T7E1 mutagenesis assay [48] and DNA sequencing after co-injection with *Cas9* mRNA into one-cell-stage zebrafish embryos (Supplemental Fig. 1a-c). We next constructed a CRISPR/Cas9-based vector system containing a tissue-specific promoter and a gene-specific target sequence [49] to spatially disrupt the expression of specific genes in zebrafish (Fig. 1a). Two key elements were inserted into a cloning vector (*Cas9-P2A-mCherry,gRNA-U6*) [50]: a zebrafish *U6–3* promoter-driven gRNA, which ensured specific expression of the targeting gRNA [51], and the *Ubb* regulatory promoter, which controlled the expression of zebrafish codon-optimized *Cas9* and the fluorescent *mCherry* reporter. Therefore, we cloned a Driver vector and a Responder vector, respectively. The Driver vector contains a liver-specific *fabp10* regulatory element, controlling the expression of Cre, which in turn efficiently transactivates the expression of the elements downstream of the *loxp-stop-loxp* cassette on the Responder vector. This approach allowed the specific expression of the downstream elements of the *loxp-stop-loxp* cassette in the presence of Cre expression to avoid the potential side effects of systemic suppressor gene mutation in zebrafish. Transgenic founder fish were generated by injecting these vectors and *Tol2* transposase mRNA (Fig. 1b), which can be translated into an active transposase in embryonic cells to catalyse the integration of a vector into the zebrafish genome [52]. The mosaic mCherry-labelled Cas9-expressing embryos were subsequently detected after the injected vector integrated randomly into a subset of embryonic cells in founder fish embryos at 48 hpf (Supplemental Fig. 1d). Thereafter, several phenotypes were identified in the offspring of the outcross between founder and WT fish, and fish with the typical phenotype that expressed consistent and robust fluorescence in liver tissue (Type-2) were selected for the generation of F_2_ transgenic fish lines (Supplemental Fig. 1e). After the incross of the Type-2 phenotype of each F_1_ transgenic fish line, the stable F_2_ lines, including the homozygous *Tg(fabp10:Cas9-mCherry);ptena*^*−/−*^ (*ptena* KO), *Tg(fabp10:Cas9-mCherry);ptenb*^*−/−*^ (*ptenb* KO), *Tg(fabp10:Cas9-mCherry);tp53*^*−/−*^ (*tp53* KO), and *Tg(fabp10:Cas9-mCherry)* (*fabp10*^WT^), were identified by fluorescence imaging at 3 dpf (Fig. 1c). To confirm the colocalization of endogenous Fabp10 and mCherry in these transgenic fish lines, we then performed immunofluorescence staining with an anti-Fabp10 antibody. The expression of endogenous Fabp10 and mCherry completely overlapped in liver tissue in the F_2_ transgenic fish line (Fig. 1d), indicating that mCherry was concomitantly expressed with *fabp10* promoter-driven Cas9 in these zebrafish.

### Identification of tissue-specific gene disruption in zebrafish

**Fig. 2:**
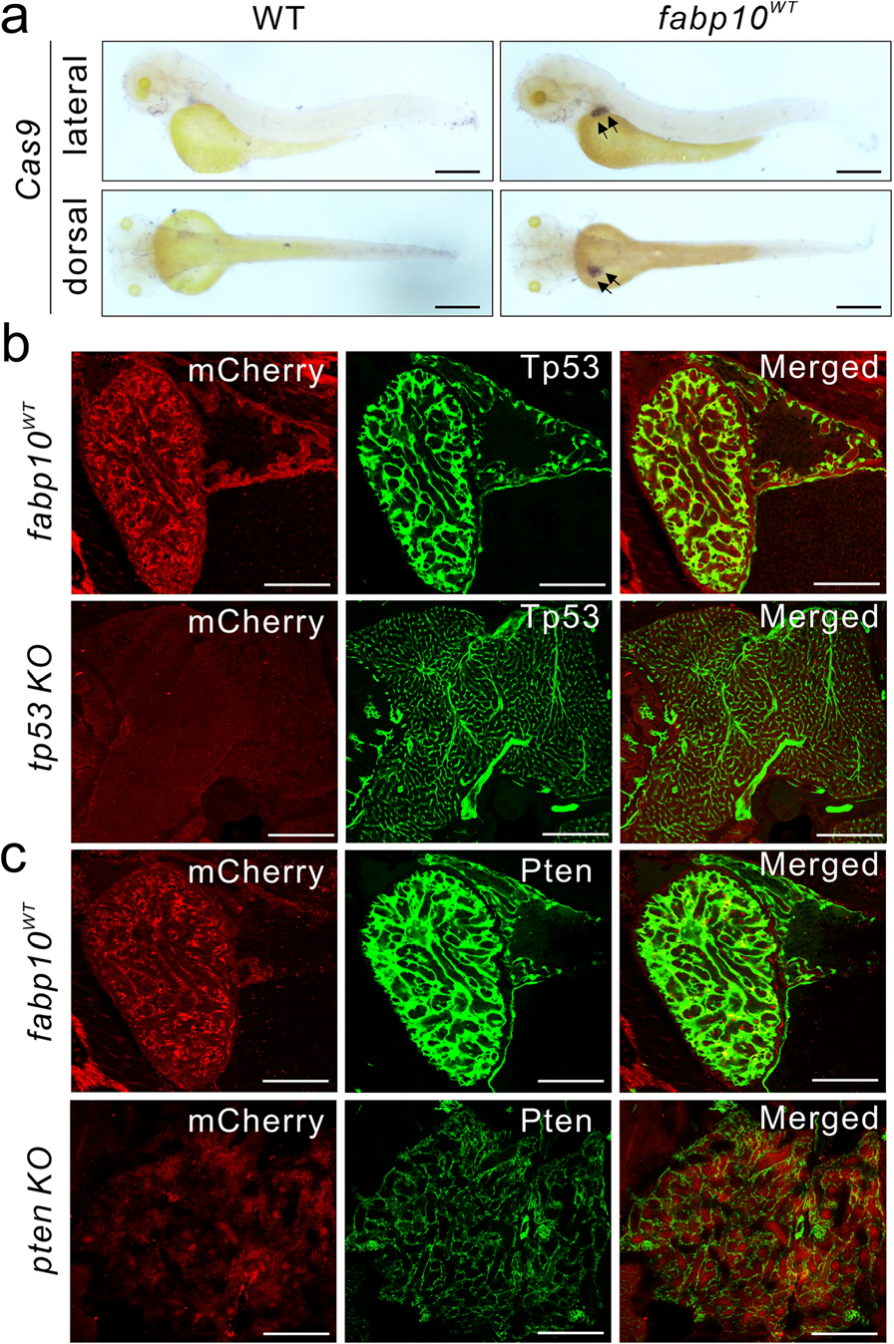
Disruption of tissue-specific Pten or Tp53 expression in larvae and mature zebrafish. **a** Representative images of whole-mount in situ hybridization using an anti-sense RNA probe against *Cas9* mRNA in 3 dpf WT and *fabp10*^*WT*^ larvae (black arrows). Scale bars, 500 μm. **b, c** Representative images of mCherry (red) and endogenous Tp53 or Pten (green, FITC-labelled) expression in liver tissues of 1-month-old *tp53* KO, *pten* KO, and *fabp10*^WT^ control fish. Scale bars, 50 μm

To verify the mutation efficiency in these transgenic fish lines, we performed DNA sequencing of randomly selected PCR products of the targeted loci. The different sequences of mutations, and the mutation indexes, which were calculated as the numbers of mutated alleles divided by the total number of sequenced alleles [49], were shown in Supplemental Fig. 2a, indicating that high-efficiency mutations were identified in *ptena* KO, *ptenb* KO and *tp53* KO fish lines. In addition, whole-mount in situ hybridization was performed to determine the mRNA expression patterns of *Cas9* in 3 dpf WT and *fabp10*^*WT*^ larvae (arrows; Fig. 2a), as well as in *tp53*, *ptena* and *ptenb* KO larvae (arrows; Supplemental Fig. 2b).

We then generated a homozygous *pten* KO fish line by incrossing *ptena* and *ptenb* KO fish (Supplemental Fig. 2c) and verified the genotype with a T7E1 mutagenesis assay (data not shown). Immunofluorescence staining was performed to confirm the tissue-specific disruption of Pten or Tp53 expression in the corresponding transgenic fish lines at 1 month post fertilization (mpf). The expression of Tp53 and Pten was significantly suppressed in 1-month-old *pten* KO and *tp53* KO fish, respectively (Fig. 2b, c). Moreover, the expression patterns of *fabp10* promoter-driven mCherry-labelled *Cas9* and endogenous Pten or Tp53 were not colocalized in these two transgenic fish lines. In contrast, in the *fabp10*^*WT*^ control group, identical colocalization between mCherry and Tp53/Pten expression was observed in liver tissues (Fig. 2b, c), indicating that the expression of endogenous targeted genes—*pten* and *tp53*—was specifically disrupted in liver tissues of *tp53* and *pten* KO fish at 1 mpf. Notably, we also found that the architectures of liver tissues were significantly disrupted in 1-month-old *pten* and *tp53* KO fish (Fig. 2b, c), suggesting that the mutation of *pten* or *tp53* might play a role in hepatic development and hepatocarcinogenesis in zebrafish.

### Pten loss induces low-grade HCC in mature zebrafish

**Fig. 3:**
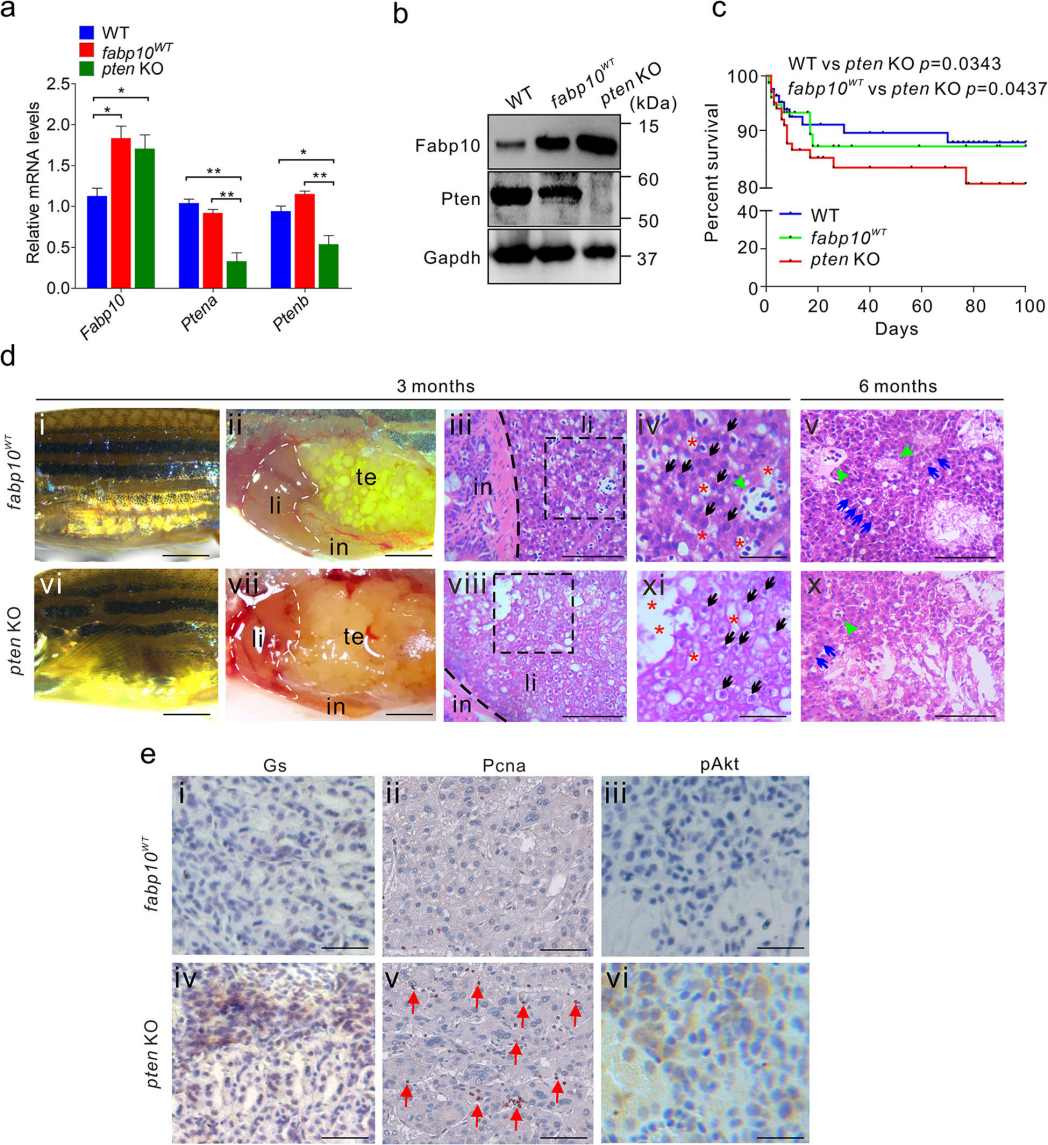
Pten mutagenesis in liver tissue disrupts liver morphology and initiates hepatocarcinogenesis. **a** Abundances of *Ptena*, *Ptenb*, and *Fabp10* mRNA in liver tissues of WT, *fabp10*^WT^, and *pten* KO fish (*n* = 3 per group). **b** Western blot analyses of Pten and Fabp10 expression in the liver tissues of WT, *fabp10*^WT^, and *pten* KO fish (*n* = 3 per group). **c** Overall survival rates of WT, *fabp10*^WT^, and *pten* KO fish (*n* = 200 per group). **d** Gross morphology of 3-month-old *fabp10*^WT^ (di) and *pten* KO fish (dvi). Representative bright field images of the internal abdominal organs, with the liver outlined, in 3-month-old *fabp10*^WT^ (dii) and *pten* KO fish (dvii). in, intestine; li, liver tissues, sb, swim bladder. Histological examination of the liver tissues from *fabp10*^WT^ (diii-dv) and *pten* KO fish (dviii-dx) at 3 and 6 months of age, respectively. Several typical hepatocarcinogenesis phenotypes were observed in 3- and 6-month-old *pten* KO fish, including abnormal lipid accumulation in hepatocytes (broken black boxes; Fig. 3diii, 3dviii), hepatocytes abnormal (black arrows; div, ix), disordered hepatic plates (blue arrows; dv, x), and veins disappearance (green arrowheads; dv, x). Scale bars, 100 μm. **e** Immunohistochemical staining was performed to examine the expression of several key tumour-related factors, including Gs, Pcna, and pAkt, in liver tissues from 3-month-old *fabp10*^WT^ and *pte*n KO fish. Scale bars, 100 μm. The data are shown as the mean ± SEM values. *p < 0.05, **p < 0.01

To further investigate the correlation between the inactivation of Pten signalling and hepatocarcinogenesis in zebrafish, we firstly evaluated the expression of Pten and Fabp10 in liver tissues of 1 mpf *pten* KO and *fabp10*^*WT*^ fish. In liver tissues of 1-month-old *pten* KO fish, the total expression of Ptena and Ptenb, which can be simultaneously recognized by the anti-Pten antibody, was significantly silenced, whereas Fabp10 expression was upregulated (Fig. 3a, b). Notably, although the survival rate of *pten* KO fish was not substantially reduced (Fig. 3c), abdominal phenotypic defects were observed in approximately 30% of *pten* KO fish (*n* = 110) at 3 months of age (Fig. 3d). The results showed that *fabp10*^*WT*^ fish exhibited normal liver tissue morphology (Fig. 3di, dii), liver hyperplasia was observed in 3 mpf *pten* KO fish (Fig. 3dvi, dvii). In addition, although clear boundaries were determined in liver tissues attached to the surface of the intestine in these two groups (broken black lines; Fig. 3diii, dviii), the lipid content in hepatocytes was significantly increased in the liver parenchyma of *pten* KO fish (broken black boxes; Fig. 3diii, dviii). In the *fabp10*^*WT*^ group, hepatocytes characterized by large and round nuclei (black arrows; Fig. 3div) were typically located near the hepatic sinuses (red asterisks; Fig. 3div), and the hepatic plates were composed of a double row of hepatocytes (blue arrows; Fig. 3dv) distributed around the veins (green arrowheads; Fig. 3div, dv). In contrast, in addition to cytoplasmic lipid accumulation in primary carcinoma tissue, 3 mpf *pten* KO fish showed more prominent liver overgrowth with pale tan, firm liver tissue (Fig. 3dvii) exhibiting spongiosis hepatitis and prominent nuclei (Fig. 3dviii-dx), consistent with the typical histopathological characteristics of primary HCC [31, 53].

Immunohistochemistry was then performed to examine the expression of several tumour-related genes in liver tissues of 6 mpf *pten* KO and *fabp10*^*WT*^ fish (Fig. 3e). In this context, glutamine synthetase (Gs), a marker of hepatocellular tumours, was diffusely expressed in *pten* KO group. In addition, the presence of Pcna-positive cells indicated more rapid proliferation in *pten* KO liver tissues than in non-tumour tissues in *fabp10*^*WT*^ control fish (red arrows; Fig. 3e). Pten is the central negative regulator of PI3K/Akt pathway [54]. In *pten* KO fish, we also found that the level of S473-phosphorylated Akt (pAkt) was slightly increased in liver tissue (Fig. 3e), indicating that the deletion of *pten* induces the activation of PI3K/Akt signalling pathway in *pten* KO fish. Interestingly, in liver tissue of *tp53* KO fish (Supplemental Fig. 3a-c), no significant histopathological feature of HCC was detected even in 6-month-old fish (data not shown), suggesting that *tp53* mutation failed to induce hepatocarcinogenesis in zebrafish. Taken together, the typical phenotypes of modest proliferation and abnormal lipid accumulation in hepatocytes observed in liver tissues of *pten* KO fish suggested that single mutation of *pten* in hepatocytes was the primary initiator of hepatocarcinogenesis and resulted in histopathological features of low-grade HCC.

### Combined inactivation of Pten and Tp53 pathways accelerates hepatocarcinogenesis in zebrafish

**Fig. 4:**
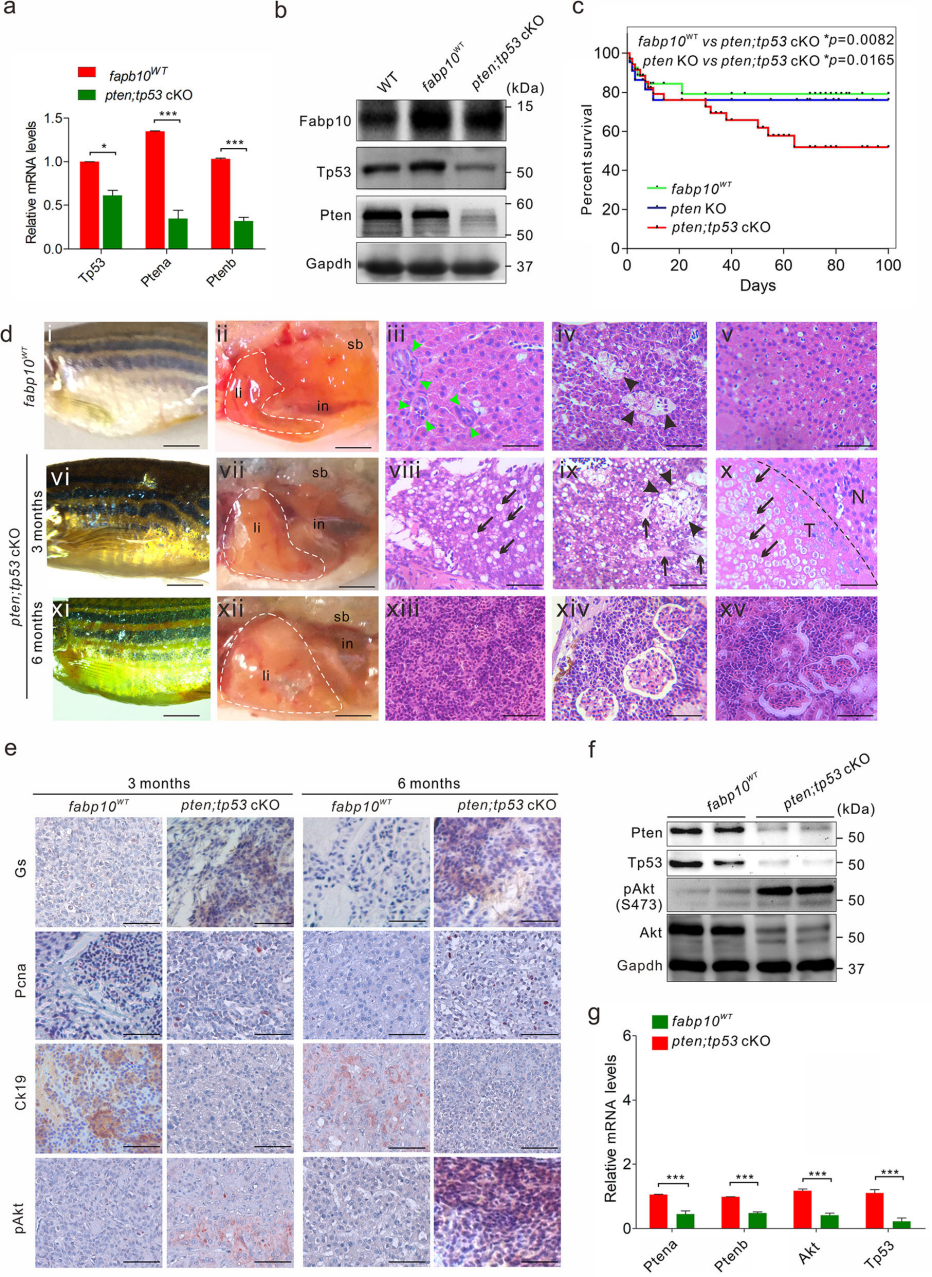
Mutation of *tp53* is critical for the progression of hepatocarcinogenesis. **a** Abundances of *Ptena*, *Ptenb*, or *Fabp10* mRNA in liver tissues of *fabp10*^WT^ and *pten;tp53* cKO fish (*n* = 3 per group). **b** Western blot analysis of Fabp10, Tp53, and Pten in liver tissues of WT, *fabp10*^WT^, and *pten;tp53* cKO fish (*n* = 3 per group). **c** Overall survival rates of WT, *fabp10*^WT^, and *pten;tp53* cKO fish (*n* = 200 per group). **d** Gross morphology of 3-month-old *fabp10*^WT^ (di) and *pten;tp53* cKO fish (dvi) and 6-month-old *pten;tp53* cKO fish (dxi). Representative bright field images of the internal abdominal organs, with the liver outlined, in 3-month-old *fabp10*^WT^ (dii) and *pten;tp53* cKO fish (dvii) and 6-month-old *pten;tp53* cKO fish (dxii). in, intestine; li, liver tissues, sb, swim bladder. Histological examination of liver tissues from *fabp10*^WT^ and *pten;tp53* cKO fish at 3 and 6 months of age, respectively. Several typical hepatocarcinogenesis phenotypes were observed in 3- and 6-month-old *pten;tp53* cKO fish, including abnormal lipid accumulation in hepatocytes (black arrows, dviii), bile duct disappearance (green arrowheads, dviii-x), vascular disorder (black arrowheads, d9), variation in nuclear/cellular sizes (pleomorphism; dvii, viii), and tumour cell invasion into blood vessels (dxiii), the pancreas (dxiv), and the kidney (dxv). Scale bars, 100 μm. **e** Immunohistochemical staining was performed to examine the expression of Gs, Pcna, Ck19, and pAkt in liver tissues from 3- and 6-month-old *fabp10*^WT^ and *pten;tp53* cKO fish. Scale bars, 100 μm. **f** Western blot analyses were performed to confirm the expression of Pten, Tp53, pAkt, and Akt in liver tissues from 3-month-old *fabp10*^WT^ (lanes 1–2) and *pten;tp53* cKO fish (lanes 3–4). **g** Quantitative analysis of the protein expression ratio of pAkt and Akt in liver tissues from 3-month-old *fabp10*^WT^ and *pten;tp53* cKO fish. The data are shown as the mean ± SEM values. *p < 0.05, ***p < 0.001

**Fig. 5:**
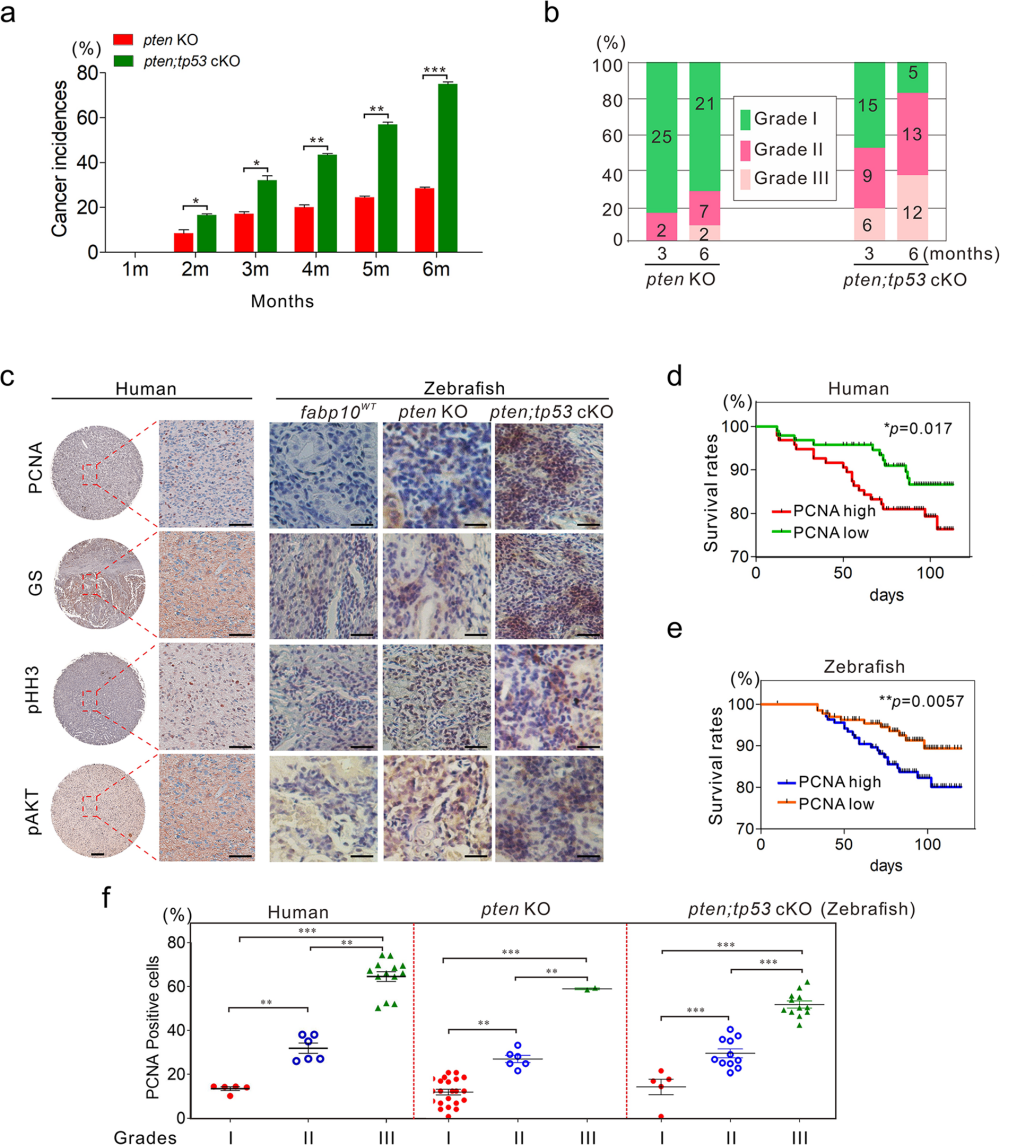
Histological and prognostic comparison of hepatocarcinogenesis between zebrafish and humans. **a** Tumour incidence rates in *pten* KO and *pten;tp53* cKO fish (*n* = 100 per group with triplicates). The tumour incidence rates were confirmed by histological examination of dissected *pten* KO and *pten;tp53* cKO fish at different time points. **b** Quantification of the malignancy of the tumours derived from fish with various mutations at 3 or 6 months of age. **c** Representative histological images indicating the expression of GS, PCNA, pHH3, and pAkt in tumours derived from humans and zebrafish with different mutations (*fabp10*^*WT*^, *pten* KO and *pten*;*tp53* cKO fish). Scale bars, 100 μm. **d, e** PCNA expression was correlative with prognosis in HCC patients (*n* = 97, d) and *pten;tp53* cKO fish (*n* = 100, e). **f** Correlation between PCNA expression and malignancy grade in HCC patients (*n* = 97), *pten* KO (*n* = 30), and *pten;tp53* cKO fish (*n* = 28). The data are shown as the mean ± SEM values. *p < 0.05, **p < 0.01, ***p < 0.001

To gain insight into the crosstalk between Tp53 and Pten signalling pathways during hepatocarcinogenesis in zebrafish, the heterozygous *pten;tp53* combined knockout (cKO) line was generated by crossing the homozygous *pten* and *tp53* KO fish lines. The expression of both Pten and Tp53 was significantly inhibited in liver tissues of 1 mpf *pten;tp53* cKO fish (Fig. 4a, b). In addition, compared with *fabp10*^*WT*^ and *pten* KO fish, the heterozygous *pten;tp53* cKO offspring (*n* = 262) showed obvious mortality at 1 month of age, and approximately 50% mortality by 2 months of age (Fig. 4c), suggesting that HCC formation, not developmental disruption, was the main cause of death in *pten;tp53* cKO fish. In addition, hepatocarcinogenesis was observed in 34% (36/106) and 73% (24/33) of liver tissues dissected from *pten;tp53* cKO fish at 3 and 6 months of age, respectively (Fig. 5a). In this context, unlike in the *fabp10*^*WT*^ control group (Fig. 4di-dv), the histopathological features of HCC, including liver hyperplasia (Fig. 4dvii), disappearance of the bile ducts (green arrowheads; Fig. 4diii, dviii), abnormal lipid accumulation in hepatocytes (black arrowheads; Fig. 4dviii, dx), vascular disorders (black arrows; Fig. 4dix), and necrosis (black arrows; Fig. 4dix), were determined in 3-month-old *pten;tp53* cKO fish. Notably, in liver tissues of 6-month-old *pten;tp53* cKO fish, even more phenotypic characteristics of high-grade HCC were observed, including haemorrhage and swollen bodies (Fig. 4dxii) and invasion of tumour cells into blood vessels (Fig. 4dxiii), the pancreas (Fig. 4dxiv), and the kidney (Fig. 4dxv).

Further immunohistochemical analyses revealed that the numbers of Gs-, Pcna-, and pAkt-positive liver cells were diffusely increased throughout the liver tumours in 3- and 6-month-old *pten;tp53* cKO fish (Fig. 4e). In addition, the immunoreactivity of cytokeratin 19 (Ck19), a marker of biliary lineage cells, was significantly decreased in *pten;tp53* cKO fish (Fig. 4e), suggesting that the absence of biliary ducts in liver tumours excluded the origin of biliary lineage cells, consistent with the symptoms of HCC in humans. Similarly, western blot analyses indicated that Akt signalling pathway was activated in randomly selected liver tissues dissected from 3-month-old *pten;tp53* cKO fish (Fig. 4f, g), suggesting that the activation of Akt pathway plays an important role in Tp53-enhanced hepatocarcinogenesis in zebrafish. Therefore, these evidence demonstrated that the cancer incidence rates and malignancy grades in *pten;tp53* cKO fish were much higher than those in *pten* KO fish, indicating that the cooperation between *tp53* and *pten* double cassette mutations significantly promotes hepatocarcinogenesis in zebrafish.

### Comparison of hepatocarcinogenesis between zebrafish and humans

To further study the similarity of hepatocarcinogenesis between zebrafish and humans, we next defined the main features, including the mortality rates, cancer incidence rates, and malignancy grades, in these transgenic fish lines with various mutational spectra (Fig. 4c, Fig. 5a, b). The cancer incidence rates and the proportions of high-grade malignancy (grades II/III) induced in *pten;tp53* cKO fish were much higher than those in *pten* KO fish (Fig. 5a, b), consistent with the trend in the mortality rates in *pten* KO, *pten;tp53* cKO, and *fabp10*^*WT*^ control fish (Fig. 4c). In this context, liver tumour formation was determined in 18 and 28% of *pten* KO fish at 3 and 6 months of age, respectively, which was much lower than the percentages of *pten;tp53* cKO fish (34 and 73%) (Fig. 5a). Similarly, the proportions of high-grade liver tumour tissues in *pten* KO fish were significantly lower than those in *pten;tp53* cKO fish. Moreover, most liver tumours (25/30) in 6-month-old *pten;tp53* cKO fish were found to exhibit histopathological features of high-grade HCC, whereas only 30% of *pten* KO fish harbouring liver tumours exhibited high-grade HCC features at 6 months of age (Fig. 5b). These findings indicated that *pten* mutation alone plays an important role in the initiation of hepatocarcinogenesis and that the additional *tp53* mutation might be critical for the progression of HCC in zebrafish.

We then performed immunohistochemistry to determine the immunoreactivity of several key hepatocarcinogenesis-related factors, including GS, PCNA, pHH3, and pAkt, in liver tumours derived from humans and zebrafish. Histological determination showed that these four genes, which exhibit high expression levels in liver tumours derived from humans, were also highly expressed in liver tumours derived from 6-month-old *pten* KO and *pten;tp53* cKO fish (Fig. 5c). In addition, taking PCNA as an example [31, 44–46], high expression of PCNA was positively correlative with poor prognosis in both humans and zebrafish (Fig. 5d, e; Supplemental Table 4). Moreover, pathological analyses revealed that the higher expression level of PCNA always companied with the higher-grade malignancy of liver tumours derived from both humans and zebrafish (Fig. 5f), suggesting that the mechanisms underlying hepatocarcinogenesis might be identical in zebrafish and humans and that this zebrafish model with various mutations might be helpful for precisely predicting prognosis and malignancy in HCC patients with different mutational spectra.

### Inactivation of Akt pathway suppresses hepatocarcinogenesis in zebrafish

**Fig. 6:**
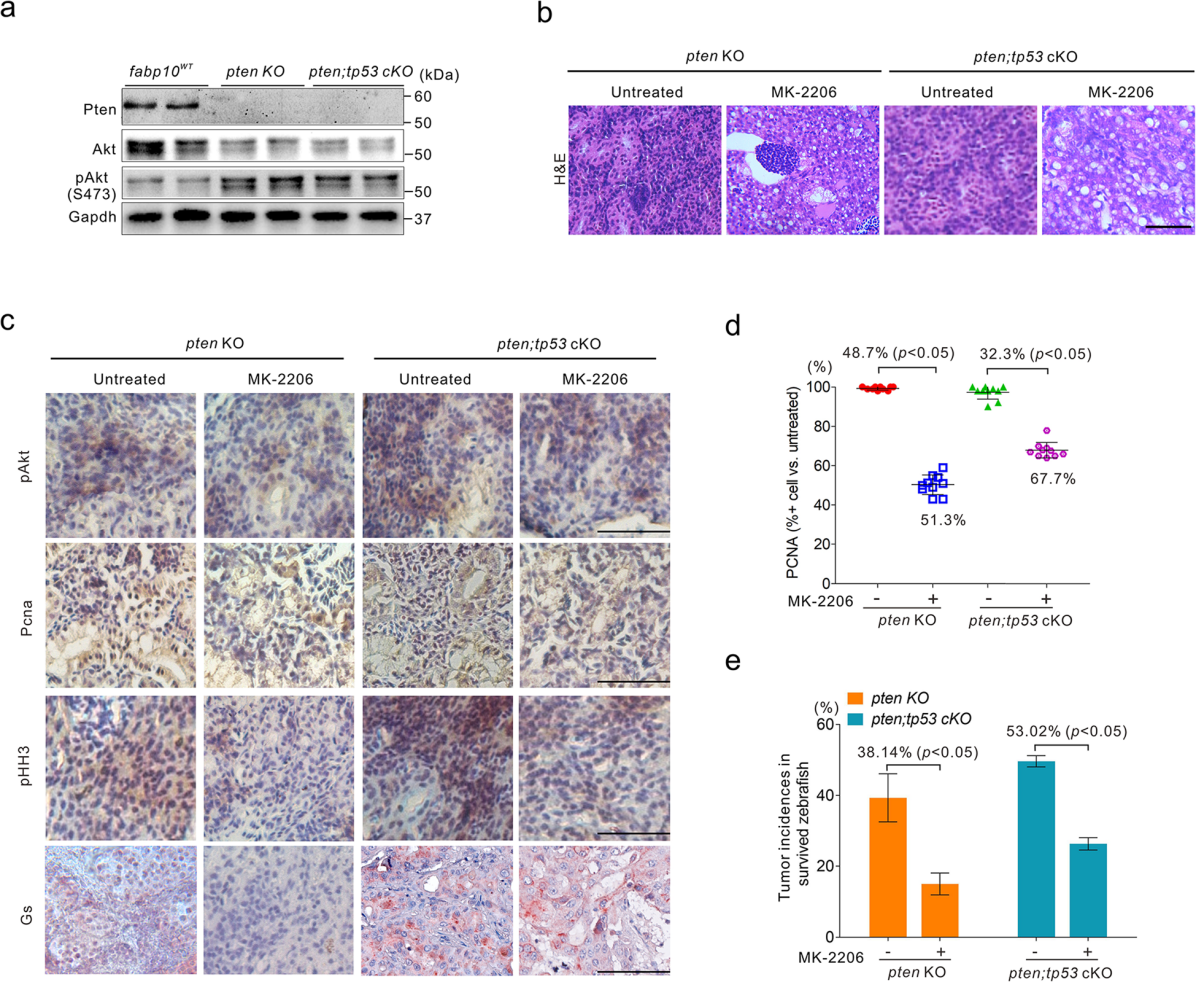
Blockade of Akt activation inhibits hepatocarcinogenesis in zebrafish. **a** Expression levels of Pten, Akt, and pAkt in liver tissues from 1-month-old *fabp10*^*WT*^, *pten* KO and *pten*;*tp53* cKO fish. **b** Representative histological images of liver tissues from *pten* KO and *pten*;*tp53* cKO fish treated with or without MK-2206. Scale bar, 100 μm. **c** Immunoreactivity of pAkt, Pcna, pHH3, and Gs in liver tissues from *pten* KO and *pten*;*tp53* cKO fish treated with or without MK-2206. Scale bars, 100 μm. **d** Quantification of the percentages of Pcna-positive cells in liver tissues from *pten* KO and *pten*;*tp53* cKO fish treated with or without MK-2206 (*n* = 10 random fields of view). **e** The tumour incidences were significantly suppressed by MK-2206 treatment in 3-month-old *pten* KO and *pten*;*tp53* cKO fish. The data are shown as the mean ± SEM values

Since the zebrafish is a popular animal model for using as a high-throughput screening platform, we next verified whether our zebrafish model can be utilized to identify antitumour compounds. Notably, the activation of Akt signalling pathway was found in liver tissues of 1-month-old *pten* KO and *pten;tp53* cKO fish (Fig. 6a), indicating that Akt pathway activation induced by the mutation of *pten* and/or *tp53* might be critical for hepatocarcinogenesis in zebrafish. We therefore treated 14 dpf larvae of *pten* KO and *pten;tp53* cKO lines with MK-2206, a specific Akt inhibitor [55], which can significantly inactivate Akt signalling pathway in zebrafish (data not shown), for up to 3 months. Histological analyses indicated a significant reduction in the malignancy grades of the dissected liver tumours in MK-2206-treated *pten* KO and *pten;tp53* cKO fish (Fig. 6b). In addition, the immunoreactivity of Gs, Phh3, Pcna, and pAkt was significantly decreased in liver tumours derived from 3-month-old *pten* KO and *pten;tp53* cKO fish (Fig. 6c). Moreover, the percentages of Pcna-positive cells indicated obvious reductions in the number of Pcna-positive proliferative cells in 3 mpf *pten* KO and *pten;tp53* cKO fish treated with MK-2206 (Fig. 6d). Importantly, we also found that the tumour incidence rates in zebrafish were effectively reduced after MK-2206 treatment (Fig. 6e). Thus, our findings suggested that these models might be helpful for hepatocarcinogenesis studies and high-throughput screening of antitumour compounds.

## Discussion

Hepatocarcinogenesis is a complex process typically involving hepatitis virus infection, non-alcoholic fatty liver disease (NAFLD), alcohol intake, autoimmune hepatitis, and chronic liver injury and inflammation [56]. Specifically, these risk factors for HCC frequently increase cancer risk and facilitate carcinogenesis by regulating multiple oncogenic molecules and signalling pathways to induce genetic alterations in hepatocytes [57]. Thus, it is important to delineate both the roles of specific frequent genetic mutations or alterations in oncogenic signalling pathways and the selective cooperativity among key tumour-related pathways in the initiation and progression of HCC [12].

Whole-genome sequencing technologies and mutational spectrum analysis approaches have led to new insights into hepatocarcinogenesis, including the identification of alterations in oncogenes and tumour suppressor genes [58, 59]. TP53 is critical for the maintenance of genome integrity by responding to various types of cellular stresses and resulting in distinct cell cycle checkpoints and resistance to apoptosis in hepatoma cells [60]. *TP53* is one of the most frequently mutated genes in human malignancies, and a previous report determined that *TP53* mutation rate was approximately 35% in HCC patients [61]. In addition, a previous study indicated that the HCV core protein can repress TP53 activity [62]. In HBx-mediated hepatocarcinogenesis, HBx not only promotes persistent HBV infection but also induces genome instability by suppressing TP53-regulating DNA repair [63], suggesting a potential correlation among *TP53* mutation, hepatitis virus infection, and hepatocarcinogenesis. Moreover, regarding β-catenin, the prevalence of β-catenin gene mutation was determined to be approximately 40% in human HCC patients [64]. Akt/PTEN signalling cascades, which are responsible for β-catenin activation, play an important role in regulating cell survival, apoptosis, and protein translation [65]. In addition, PTEN has been reported to be a crucial negative regulator of PI3K/Akt signalling pathway, and genetic and epigenetic alterations in PTEN or its regulatory regions can promote hepatocarcinogenesis [66, 67].

To achieve tissue-specific expression of *pten* and *tp53* in zebrafish, we established an HCC model in a *Cre-loxp* transgenic zebrafish line via a CRISPR/Cas9 system directly targeting tumour suppressor genes in zebrafish liver tissues through Cas9-mediated mutation of the *pten* and/or *tp53* locus under the control of the *fabp10* promoter. Our models demonstrated that single mutation of *tp53* failed to induce primary HCC in zebrafish within the first 6 months of life. In contrast, single mutation of the *pten* locus induced the activation of Akt signalling pathway but did not result in features of advanced hepatocarcinogenesis, such as abnormal lipid accumulation, steatohepatitis, and tissue necrosis, in *pten* KO fish (Fig. 3d, e). In addition, only low-grade HCC with a low tumour incidence (less than 20%) was observed in 3-month-old *pten* KO fish (Fig. 5a, b), suggesting that *pten* mutation is critical only for the initiation of HCC and that additional genetic lesions in the pathway might be required for subsequent tumour progression. Moreover, obviously elevated malignancy grade, enhanced tumour incidence, and increased histological grade and invasiveness were observed in 3-month-old *pten;tp53* cKO fish (Fig. 4d). Although *tp53* mutation alone was insufficient to initiate hepatocarcinogenesis, double mutation of *tp53* and *pten* effectively promoted the development of high-grade HCC in zebrafish (Fig. 5a, b), indicating that *tp53* mutation can contribute to HCC progression in zebrafish. It is noted that mice deficient in *tp53* are developmentally normal but susceptible to spontaneous tumours [68]. After comparison of the molecular and histological signatures in zebrafish with *tp53* and/or *pten* mutation, we hypothesized that without the protective surveillance afforded by Tp53, *pten*-deficient hepatocytes are more susceptible to the progression of HCC in zebrafish.

The mechanisms through which normal liver cells transform into hepatocellular carcinoma are highly complicated, and the specific signalling pathways might play different roles through certain arranged sequences [69]. Our results revealed that the spontaneous HCC developed when *pten* are deleted in zebrafish, which might have implications for cancer therapy, whereas the deficiency of Tp53 is not required for the initiation of HCC, indicating that Akt/Pten pathway might be critical for the initiation pathway of hepatocarcinogenesis in zebrafish. Mechanistically, it is known that PTEN, which negatively regulates AKT pathway [70, 71], is among the most frequently inactivated tumours suppressor gene in different malignant cancers. However, the previous tissue-specific *pten*-deficient animal models showed that PTEN functions on tumorigenesis were various in different tissues [72]. The activation of PTEN/Akt signalling was known to play important roles in cell cycle arrest and inhibition of cell invasion [73–75]. Several key downstream regulators of PTEN/Akt signalling, such as GSK3, CHK1, MAPK3, FOXO, were critical for tumour cell growth and survival [76]. Importantly, mTOR activity is also enhanced after the inactivation of PTEN pathway [77, 78]. In contrast, the inhibition of mTOR signalling, such as rapamycin, temsirolimus or everolimus, significantly contributes to cancer prevention [79–82]. Therefore, our evidence indicated that these downstream targets of PTEN/Akt signalling, which are mainly involved in the regulation of cell proliferation, cell invasion and cell survival, might play more important roles for the initiation of hepatocarcinogenesis. Interestingly, although the aberrations in TP53 pathway were determined in most of malignant tumours [83], the mutation of *tp53* alone could not trigger hepatocarcinogenesis in *tp53* KO fish. However, we noticed that the combination of *pten* and *tp53* was often correlative with high-grade histology and poor prognosis in zebrafish (Figs. 4, 5), suggesting that *TP53* mutation-induced the regulation of certain downstream targets, including VEGF-A, CDKN1A, BAX, IGFBP3, MDM2, FAS [84, 85], might be critical for the progression pathway of hepatocarcinogenesis, which is consistent with the previous report that *TP53* mutations in HCC patients were usually accompanied by worse clinical stage and prognosis [86].

We noted the abnormal lipid accumulation in hepatocytes in the tumours derived from liver tissues of *pten* cKO fish, and a trend towards increased hepatic steatosis in *pten;tp53* cKO fish (Figs. 3d, 4d). Our results also showed that the dual mutations of *tp53* and *pten* greatly increased Akt phosphorylation and obviously induced the features of nonalcoholic steatohepatitis (NASH), including infiltration of inflammatory cells, collagen deposition, and abnormal lipid accumulation [7, 87], in tumours derived from liver tissues in zebrafish (Fig. 4d-g). We therefore expected that the malignant phenotype of NASH might be a critical biological event during *pten/tp53*-induced hepatocarcinogenesis.

A previous report demonstrated that the global loss of Akt1/2 signalling could be pro-tumorigenic in the liver tissue in mice [88]. Considering the importance of the endogenous Pten/Akt signalling in maintain normal homeostasis [89], we think that our conclusion that the inactivation of PTEN/Akt signalling could suppressed hepatocarcinogenesis (Fig. 6), might not be conflictive with the previous report. In our study, we treated the transgenic fish with 5 μM MK-2206, which has no obvious side effects in MK-2206-treated juvenile or adult fish (> 14 dpf) in our preliminary experiments (Data not shown) and the previous report [55]. Wang et al. showed that the complete loss of Akt1/2 could induce hepatocarcinogenesis, and the deletion of one Akt1 allele with complete loss of Akt2 is insufficient to drive the appearance of HCC [88], indicating that there might be possible for the treatment of Akt inhibitor with an acceptable toxicity and minimal hepatocarcinogenesis risk. In addition, unlike genetic deletion, the temporary treatment of Akt inhibitor might further limit the potential carcinogenesis, which is consistent to the conclusion that the transiently MK-2206-treated mice have shown no obvious signs of HCC emergence [88]. It is noted that some of harmful side effects on gastrointestinal system and metabolism [90, 91] are similar with the symptoms of the complete loss of Akt1/2 in mice model [88], indicating that we might pay more conscientious for the side effects of Pten/Akt signalling as a potential anticancer therapeutic target. Moreover, the discrepancies between activation/inactivation of Pten/Akt signalling [28, 92] and the transgenic loss of Akt1/2 suggested that certain other biological events might occur in the dual deletion of Akt1/2 during hepatocarcinogenesis in mice model.

Our results indicated marked tumour regression through MK-2206-mediated inactivation of Akt signalling pathway in these transgenic fish lines (Fig. 6). The critical role of suppressive phosphorylation of Akt in hepatocarcinogenesis suggests that pharmacological targeting of PTEN/Akt pathway probably offers potential therapeutic benefits for HCC patients. There were several identified compounds targeting PTEN/Akt signalling, such as perifosine and MK-2206, could suppress tumour growth in animal models or clinical trials [90, 91, 93–97]., suggesting that the pharmacological inhibition of PTEN/Akt signalling might be a potential target for the treatment of tumour patients. Taken together, our findings might provide a cost-effective and high-throughput platform for identifying and evaluating candidate anticancer drugs, which is not feasible in mouse models. In addition, the feasibility of acquiring a toxicity profile in a physiologic context is an additional benefit of in vivo studies. Because of these inherent advantages, high-throughput screening is potentially a valuable approach for compound discovery.

## Conclusion

In summary, we systematically investigated hepatocarcinogenesis in zebrafish with *pten* and/or *tp53* KO and clarified the synergistic crosstalk between aberrant Pten and Tp53 signalling pathways during different stages of hepatocarcinogenesis. The findings revealed that Pten loss mainly plays an important role in HCC initiation and frequently induces low-grade HCC, whereas the cooperative *tp53* mutation is critical for the progression during hepatocarcinogenesis in zebrafish. In addition, histological and pathological analyses of the tumours derived from zebrafish indicated that these tumours were largely similar to those of humans, suggesting that the identical pathogenic processes occur during hepatocarcinogenesis in zebrafish and humans. The inhibitor experiments also indicated that our transgenic models might be suitable for screening anticancer drugs for the treatment of patients with HCC, which enhances the value of zebrafish as a model organism for use as a high-throughput screening platform.

## Supplementary Information

**Additional file 1: Supplemental Figure 1.** Targeted expression of transgenes in zebrafish embryos. **a**-**c** T7E1 assays and DNA sequencing were performed to determine the mutations of *tp53* (a), *ptena* (b) and *ptenb* (c) in whole embryos injected with *Cas9* mRNA and target gRNAs, respectively. The CRISPR target sequences were marked in blue PAM, and the mutations were marked in red under yellow background. **d** Mosaic founder phenotypes of *fabp10*^*WT*^ larvae at 48 hpf. **e** The different mCherry-labelled Cas expression patterns in transgenic larvae at 48 hpf. Scale bars, 500 μm. **Supplemental Figure 2.** The identification of liver-specific mutations of *tp53*, *ptena* and *ptenb* genes in zebrafish. **a** DNA sequencing were performed to evaluate the efficiency in whole embryos injected with Cas9 mRNA and target gRNAs, respectively. Red, the CRISPR target sequences; green, PAM; yellow, mutations. **b** Representative images of whole mount in situ hybridization using an anti-sense RNA probe against *Cas9* mRNA in 3 dpf *tp53*, *ptena*, and *ptenb* KO larvae. Scale bars, 500 μm. **c** The strategy of the generation of *pten* KO fish line. **Supplemental Figure 3.** The determination of liver-specific *tp53* mutation in zebrafish. **a** Abundances of *Tp53* and *Fabp10* mRNA in liver tissues of WT, *fabp10*^WT^ and *tp53* KO fish (*n* = 3 per group). **b, c** Western blot analyses and quantification of Fabp10 and Tp53 in liver tissues of WT, *fabp10*^WT^, and *tp53* KO fish (*n* = 3 per group). Data shown as mean ± SEM. **p < 0.01. **Supplemental Table 1.** The primer sequences for gRNAs synthesis and T7E1 assay. **Supplemental Table 2.** The primer sequences for qRT-PCR assay. **Supplementary Table 3.** The information of antibodies. **Supplemental Table 4.** Correlation between PCNA and clinicopathological characteristics of the patients with HCC.

## Data Availability

All data generated or analysed during this study available from the corresponding author on reasonable request.

## Abbreviations

HCC: Hepatocellular carcinoma
HBV: Hepatitis B virus
HCV: Hepatitis C virus
dpf: Days post-fertilization
mpf: Months post-fertilization
NASH: Nonalcoholic steatohepatitis
