# Supplementary material for: Cooperation between liver-specific mutations of pten and tp53 genetically induces hepatocarcinogenesis in zebrafish
Source: J Exp Clin Cancer Res. 2021 Aug 20;40:262. doi: 10.1186/s13046-021-02061-y (PMC8377946; doi:10.1186/s13046-021-02061-y)
Supplement: Supplementary file 1 — Additional file 1: Supplemental Figure 1. Targeted expression of transgenes in zebrafish embryos. a-c T7E1 assays and DNA sequencing were performed to determine the mutations of tp53 (a), ptena (b) and ptenb (c) in whole embryos injected with Cas9 mRNA and target gRNAs, respectively. The CRISPR target sequences were marked in blue PAM, and the mutations were marked in red under yellow background. d Mosaic founder phenotypes of fabp10WT larvae at 48 hpf. e The different mCherry-labelled Cas expression patterns in transgenic larvae at 48 hpf. Scale bars, 500 μm. Supplemental Figure 2. The identification of liver-specific mutations of tp53, ptena and ptenb genes in zebrafish. a DNA sequencing were performed to evaluate the efficiency in whole embryos injected with Cas9 mRNA and target gRNAs, respectively. Red, the CRISPR target sequences; green, PAM; yellow, mutations. b Representative images of whole mount in situ hybridization using an anti-sense RNA probe against Cas9 mRNA in 3 dpf tp53, ptena, and ptenb KO larvae. Scale bars, 500 μm. c The strategy of the generation of pten KO fish line. Supplemental Figure 3. The determination of liver-specific tp53 mutation in zebrafish. a Abundances of Tp53 and Fabp10 mRNA in liver tissues of WT, fabp10WT and tp53 KO fish (n = 3 per group). b, c Western blot analyses and quantification of Fabp10 and Tp53 in liver tissues of WT, fabp10WT, and tp53 KO fish (n = 3 per group). Data shown as mean ± SEM. **p < 0.01. Supplemental Table 1. The primer sequences for gRNAs synthesis and T7E1 assay. Supplemental Table 2. The primer sequences for qRT-PCR assay. Supplementary Table 3. The information of antibodies. Supplemental Table 4. Correlation between PCNA and clinicopathological characteristics of the patients with HCC. [file 13046_2021_2061_MOESM1_ESM.zip › Luo_Supplemental File_R2.docx]

**Additional file: Supplemental Figure 1.** Targeted expression of transgenes in zebrafish embryos. **Supplemental Figure 2.** The identification of liver-specific mutations of *tp53*, *ptena* and *ptenb* genes in zebrafish. **Supplemental Figure 3.** The determination of the liver-specific tp53 mutation in zebrafish. **Supplemental Table 1.** The primer sequences for qRT-PCR assay. **Supplemental Table 2.** The primer sequences for gRNAs synthesis and T7E1 assay. **Supplemental Table 3**. The information of antibodies. **Supplemental Table 4.** Correlation between PCNA and clinicopathological characteristics of the patients with HCC.

**Supplemental Figure Legends**

**Supplemental Figure 1.** Targeted expression of transgenes in zebrafish embryos. **a**-**c** T7E1 assays and DNA sequencing were performed to determine the mutations of *tp53* (a), *ptena* (b) and *ptenb* (c) in whole embryos injected with *Cas9* mRNA and target gRNAs, respectively. The CRISPR target sequences were marked in blue PAM, and the mutations were marked in red under yellow background. **d** Mosaic founder phenotypes of *fabp10^WT^* larvae at 48 hpf. **e** The different mCherry-labelled Cas expression patterns in transgenic larvae at 48 hpf. Scale bars, 500 μm.

**Supplemental Figure 2.** The identification of liver-specific mutations of *tp53*, *ptena* and *ptenb* genes in zebrafish. **a** DNA sequencing were performed to evaluate the efficiency in whole embryos injected with Cas9 mRNA and target gRNAs, respectively. Red, the CRISPR target sequences; green, PAM; yellow, mutations. **b** Representative images of whole mount *in situ* hybridization using an anti-sense RNA probe against *Cas9* mRNA in 3 dpf *tp53*, *ptena*, and *ptenb* KO larvae. Scale bars, 500 μm. **c** The strategy of the generation of *pten* KO fish line.

**Supplemental Figure 3.** The determination of liver-specific *tp53* mutation in zebrafish. **a** Abundances of *Tp53* and *Fabp10* mRNA in liver tissues of WT, *fabp10*^WT^ and *tp53* KO fish (n = 3 per group). **b, c** Western blot analyses and quantification of Fabp10 and Tp53 in liver tissues of WT, *fabp10*^WT^, and *tp53* KO fish (n = 3 per group). Data shown as mean ± SEM. ***p* < 0.01.

**Supplemental Tables**

**Supplemental Table 1.** The primer sequences for gRNAs synthesis and T7E1 assay.

| **Genes** | **Sequences of primers** | |
| --- | --- | --- |
| *T7-ptena target site* | F: 5’-TAATACGACTCACTATAGGGAGGGTTGTGGTCCTCGAAGTTTTAGAGCTAGAAATAGC-3’ |  |
|  | R: 5’-AGCACCGACTCGGTGCCAC-3’ |  |
| *T7E1-ptena* | F: 5’-TTGCCACTTGTTTCACACAGA-3’ |  |
|  | R: 5’-AATGCAGCAACAGTTCCAGT-3’ |  |
| *T7-ptenb target site* | F: 5’-TAATACGACTCACTATAGGGAGTGACTATTCCCAGTCAGGTTTTAGAGCTAGAAATAGC-3’ |  |
|  | R: 5’-AGCACCGACTCGGTGCCAC-3’ |  |
| *T7E1-ptenb* | F: 5’-ACACAAGTGCTGTCCCCTTT-3’ |  |
|  | R: 5’-CATTGTTGGCACAGACGCAAGCTCATA-3’ |  |
| *T7-tp53 target site* | F: 5’-TAATACGACTCACTATAGGGTGGACGTTGCCCCTCCACGTTTTAGAGCTAGAAATAGC-3’ |  |
|  | R: 5’-AGCACCGACTCGGTGCCAC-3’ |  |
| *T7E1-tp53* | F: 5’-GGAGCCACTTGAGGTTGAGT-3’ |  |
|  | R: 5’-TTGCCTCCCTCGCCATAGTA-3’ |  |

**Supplemental Table 2.** The primer sequences for qRT-PCR assay

| **Genes** | **Sequences of primers** | |
| --- | --- | --- |
| *RT-ptena* | F: 5’-CGAACACCATCGATCGCAGA-3’ |  |
|  | R: 5’-CTGACCCGGATGAAGACAGG-3’ |  |
| *RT-ptenb* | F: 5’-TACAAACCTGTGGCCTTGCT-3’ |  |
|  | R: 5’-CACCTTGATGTCCCCACACA-3’ |  |
| *RT-fabp10* | F: 5’-AACTCCTTCACCATCGGCAA-3’ |  |
|  | R: 5’-ATCATGGTGGTTCCTCCGAC-3’ |  |
| *RT-akt1* | F: 5’-TAAGGAGCGACCGCAAGATG-3’ |  |
|  | R: 5’-TGCAGGCAGCGTATGATGAA-3’ |  |
| *RT-pcna* | F: 5’-TGTTTGAGGCACGTCTGGTT-3’ |  |
|  | R: 5’-TAGGAGTCGAACCCGTCACT-3’ |  |
| *RT-tp53* | F: 5’-CACCACTGGGACCAAACGTA-3’ |  |
|  | R: 5’-GTCGCTTCTTCCTTCGTCCT-3’ |  |
| *RT-actin* | F: 5’-CCCTGAATCCCAAAGCCAA-3’ |  |
|  | R: 5’-CCATCACCAGAGTCCATCACAATA-3’ |  |

**Supplementary Table 3**. **The information of antibodies.**

| **Antibodies** | **Suppliers** | **Catalog Number** | **Concentrations** | **Experiments** |
| --- | --- | --- | --- | --- |
| Anti-Pten antibody | LSBio | LS‑C368655 | 1:2000 / 1:100 | Western blot / IF |
| Anti-Ck19 antibody | LSBio | LS-B3148 | 1:100 | IHC |
| Anti- phospho-Akt antibody | Cell Signaling Technology | 4060S | 1:100 | IHC |
| Anti Fabp10 antibody | BioSciences | C344616 | 1:100 | IF |
| Anti Fabp10 antibody | Invitrogen | PA5-72183 | 1:1000 | Western blot |
| anti-Gs antibody | Abcam | AB64613 | 1:100 | IHC |
| Anti-Tp53 antibody | Abcam | AB77813 | 1:2000 / 1:200 | Western blot / IF |
| Anti-Akt antibody | Abcam | AB227100 | 1:2000 | Western blot |
| Anti-Pcna antibody | Abcam | AB220208 | 1:200 | IHC |
| Anti-pHH3 antibody | Cell Signaling technology | 9764S | 1:200 | IHC |
| Anti- phospho-Akt antibody | Santa Cruz | SC-52940 | 1:2000 | Western blot |
| Anti-Gapdh antibody | Abcam | AB8245 | 1:2000 | Western blot |

**Supplemental Table 4.** Correlation between PCNA and clinicopathological characteristics of the patients with HCC.

| Characteristics | Cases | PCNA expression | | *P* |
| --- | --- | --- | --- | --- |
|  |  | **High (%)** | **Low (%)** |  |
| *Age (years)* |  |  |  | 0.0721 |
| *< 50* | 13 | 6 (46.2) | 7 (53.8) |  |
| *≥ 50* | 11 | 9 (81.8) | 2 (18.2) |  |
| *Gender* |  |  |  | 0.0528 |
| *Male* | 20 | 5 (25) | 15 (75) |  |
| *Female* | 4 | 3 (75) | 1 (25) |  |
| *Tumor Grades* |  |  |  | 0.0150 |
| *I+II* | 11 | 3 (27.3) | 8 (72.7) |  |
| *III* | 13 | 10 (76.9) | 3 (23.1) |  |
